# Fundamental Interaction Niches: Towards a Functional Understanding of Ecological Networks' Resilience

**DOI:** 10.1111/ele.70146

**Published:** 2025-05-30

**Authors:** Emma‐Liina Marjakangas, Bo Dalsgaard, Alejandro Ordonez

**Affiliations:** ^1^ Section for Ecoinformatics and Biodiversity, Department of Biology Aarhus University Aarhus Denmark; ^2^ Center for Ecological Dynamics in a Novel Biosphere (ECONOVO), Department of Biology Aarhus University Aarhus Denmark; ^3^ Section for Molecular Ecology and Evolution Globe Institute, University of Copenhagen Copenhagen Denmark; ^4^ Center for Sustainable Landscapes Under Global Change (SustainScapes), Department of Biology Aarhus University Aarhus Denmark

**Keywords:** adaptability, antagonistic interaction, functional diversity, fundamental niche, interaction rewiring, metanetwork, mutualistic interaction, pairwise interaction, realised niche

## Abstract

Global change will create new species interactions and alter or eliminate existing ones, a process known as interaction rewiring. This rewiring can significantly affect how ecosystems function. To better predict the future structure of ecological networks, assessing their ability to adapt to changes is crucial. Here, we introduce two concepts: ‘rewiring capacity’ of a single species (the multidimensional trait space of all its potential interaction partners within a region) and ‘rewiring potential’ of a local community (the total trait space covered by interaction partners of the species at the target trophic level locally). To quantify the rewiring capacity and potential, we apply existing methods for determining species' functional interaction niches in a novel way to assess species' and communities' ability to form new interactions and the functional resilience of interaction networks to global change. To illustrate the applicability of these concepts, we quantified the rewiring capacity and potential of interactions between 1002 flowering plant species and 318 hummingbird species across the Americas. The rewiring capacity and potential metrics offer a new way to understand and quantify network resilience, allowing us to map how ecological networks respond to global change.

## Graphical abstract

Global change pressures can reshuffle community compositions and reorganise structures of ecological networks. This paper provides concepts and tools to assess the functional ability of species and networks to reorganise their interactions under global change. The use of the concepts and tools is illustrated with a case study on flowering plant–hummingbird networks in the Americas.

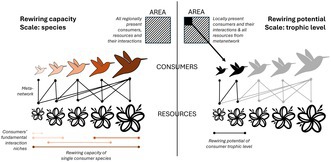

## 1 Introduction

In the face of global change, species' distributions are shifting to higher altitudes and latitudes as organisms track their preferred climatic conditions, altered by increasing temperatures and changing precipitation patterns (Chen et al. 2011). This movement disrupts existing ecological balances, reshuffling community compositions and potentially resulting in the emergence of novel communities (Blowes et al. 2019; Marjakangas et al. 2023). Such changes inevitably lead to the reorganisation of ecological networks, where interactions among species—such as predation, competition, and mutualism—are lost, altered or newly established, with potentially far‐reaching consequences for ecosystem structure and function (Bartley et al. 2019). The reorganisation of species interactions may contribute to determining a network's resilience, broadly defined as the ability to resist change under perturbation and return to equilibrium following disturbance (Thébault and Loreau 2005). Thus, network resilience is not just a function of the diversity of species present but also of the complexity and redundancy of their interactions, which could buffer the networks against the impacts of species losses or invasions (Dunne et al. 2002). As global changes continue to drive shifts in species distributions and community structures, the resilience of networks—how well they can maintain their functions and services in the face of such changes—becomes a pivotal factor in determining long‐term ecological outcomes.

Species interactions in ecological networks contribute to a wide range of critical ecosystem functions and services, such as plant reproduction and carbon storage (mutualistic plant‐pollinator and seed‐dispersal interactions), pest control (antagonistic predator–prey interactions), and disease control (commensalistic carcass removal interactions; Harvey et al. 2017; IPBES 2016). Importantly, the ecological networks of pairwise interactions underpin how ecosystem functions vary in their resilience and capacity to adjust to changing environmental conditions. Here, we define network resilience as the disturbance that can be tolerated before a system shifts to a different equilibrium state (Carpenter et al. 2001). In the specific context of ecological networks under global change, resilience is the maintenance of ecological functions even when there is a global change‐driven turnover in pairwise interactions. For example, we consider a pollination network resilient if all its plant species continue to be pollinated in a situation where some pollinator species go locally extinct or new ones establish in the community.

**FIGURE 1:**
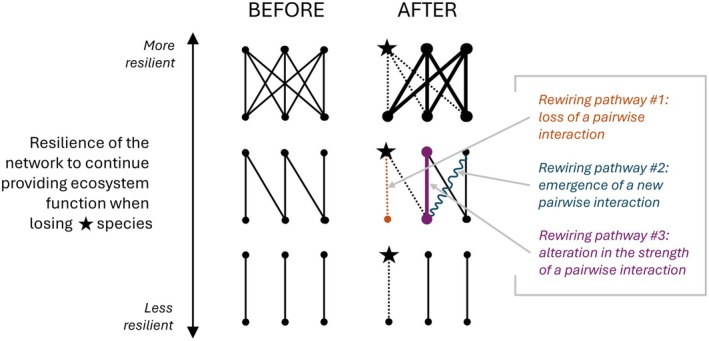
Schematic illustration of variation in network's resilience (y‐axis arrow) to losing one species at the higher trophic level in the network (indicated with a black star in the top‐left of each network) in the context of interaction rewiring. In the illustrated networks, pairwise interaction links (lines) occur between three consumer species at a higher trophic level (upper points) and three resource species at a lower trophic level (lower points). The temporal change in interactions (difference in links between BEFORE and AFTER) is illustrated with dashed (lost interaction), bolded (strengthened interaction), and wavy (emerged interaction) lines, each of which represents one of the possible ways that a pairwise interaction link can be rewired.

Resilience of a network can arise from the reorganisation of pairwise interactions (Vizentin‐Bugoni et al. 2020; Watts and Strogatz 1998), termed ‘interaction rewiring’ (Figure 1). Interaction rewiring can occur via different pathways: #1 loss of existing interactions between species, #2 the emergence of new interactions, and #3 alterations in the strengths of the interactions between species (topological vs. interaction strength rewiring; Vizentin‐Bugoni et al. 2020; Watts and Strogatz 1998; Figure 1). Such rewiring happens due to the turnover of species composition and/or the rearrangement of interactions among species continuously present in the community over time (e.g., seasonal rewiring, Brimacombe et al. 2021). For example, when interaction rewiring occurs in a local pollination network, a new pollinator takes over the role of one that disappeared or whose abundance declined following an environmental perturbation. Thus, the pollination ecosystem function is secured despite the turnover in the identity of the pollinator species providing it (Schrøder et al. 2024). In an alternative scenario, the role of a lost pollinator species cannot be filled by an existing or a newly colonising species, leaving the role vacant in the pollination network. More broadly, if species can rewire their interactions, the networks' resilience increases, for instance, to global change drivers impacting biodiversity (e.g., climate change, Schleuning et al. 2016; Sonne et al. 2022). Therefore, whether the pairwise interactions can be rewired could drastically affect the ecosystems and the future provision of nature's functions and services to humanity (IPBES 2016).

Niche theory helps to explain species' relationships to their abiotic environment (Grinnellian niches) and to their biotic environment, i.e., the ecological interactions and the species' roles in the local network (Eltonian niches) (Soberón and Nakamura 2009). An interaction niche describes the identity and functional properties of the partners with which a species can interact (Albrecht et al. 2018; Dehling et al. 2016; Ponisio et al. 2019). The sizes of a species' environmental and interaction niches indicate the breadth of abiotic and biotic conditions the species can tolerate. Following niche theory, it can be assumed that a species with a large abiotic and/or biotic niche is more resilient to disturbances, such as those caused by global change (Comte et al. 2024), because it can adjust its resource use to new conditions more easily than a species with a limited abiotic and/or biotic niche.

Despite the urgency of assessing the resilience of ecosystem functioning that builds on species interactions, major knowledge gaps in species' and networks' capacity to adapt to future environmental changes remain. Although species extinctions and decreasing abundances affect networks via topological and interaction strength rewiring (Bartley et al. 2019; Dunne et al. 2002), the current static approaches fail to consider the consequences of ecological network rewiring due to the lack of discourse between network ecology and projections of community changes. Notably, current practices either document rewiring and network robustness to species loss empirically at small spatiotemporal scales (Arroyo‐Correa et al. 2023; Costa et al. 2018; Montero‐Castaño and Vilà 2017; Polazzo et al. 2022; Schrøder et al. 2024; Staniczenko et al. 2010) or theoretically from species removal exercises (Dunne et al. 2002; Memmott et al. 2004; Timóteo et al. 2016) most often without accounting for the emergence of new interactions (but see Sonne et al. 2022). Indeed, forecasts of species' interactions in the future rarely include colonisations of species, limiting the scenarios to be based on predicted extinctions only. At broad scales, we do not know if the capacity of networks to rewire varies among ecosystems, whether species dispersing into new ecosystems will result in the rewiring of existing networks, or how we should even quantify rewiring and its potential at broad spatial, temporal and taxonomic scales. Thus, more studies that account for the future colonisations of range‐shifting and human‐introduced species are needed (Vizentin‐Bugoni et al. 2020). Moreover, to forecast how networks will look and function in the future, we need to know the limits of their functional potential.

In this perspective, we aim to combine interaction niche theory and trait‐based approaches to introduce the concepts of species‐specific rewiring capacity and trophic level‐specific rewiring potential. We develop these novel concepts by synthesising resilience theory, fundamental niches, species' functional roles, and interaction rewiring (see next section). We illustrate the concepts with an empirical dataset of interactions between flowering plants and hummingbirds in mutualistic pollination networks in the Americas and show how the species‐specific and trophic level‐specific proxies of resilience can be quantified in practice. Our conceptual framework provides a mechanistic understanding of the functional basis of network resilience against global change.

## 2 A Conceptual Framework for Understanding Networks' Resilience Using Functional Interaction Niches

### 2.1 Trait‐Based Approaches in Network Ecology

Both species' interactions and ecosystem functions can be studied with trait‐based approaches, as species' characteristics determine their functional roles in the network and the ecosystem beyond their taxonomic identities (Albrecht et al. 2018; Sobral et al. 2023). According to theoretical and empirical studies, trait‐based assembly rules govern the presence and the potential for pairwise interactions between species (Albrecht et al. 2018; Chacoff et al. 2018; Fricke et al. 2022; Marjakangas et al. 2022; Morales‐Castilla et al. 2015) and interaction rewiring (Vizentin‐Bugoni et al. 2020). For example, functional matching governs the presence and the strength of pairwise interactions, such that long‐billed hummingbirds often visit flowers with long corollas (Dalsgaard et al. 2021). Thus, a theoretical and empirical basis exists for trait‐based assessment of interaction rewiring.

### 2.2 Functional Interaction Niches

Traditionally, species' interaction niches and roles in the networks have been studied either by identifying the taxonomic identities and numbers of interaction partners (Schleuning et al. 2016) or by using the functional or evolutionary uniqueness of the focal species as a predictor of its interactions with species at another trophic level (Pigot et al. 2016). Moving from interaction partner identities to functional trait‐based characteristics establishes an ecologically relevant link from interactions to ecosystem functioning (Dehling et al. 2016). For example, rather than identifying the specific prey species that a predator consumes, assessing the size distribution of the prey species will shed light on the functional role of the predator participating in the top‐down regulation of the food web (Eklöf et al. 2013; Pires 2024). Trait‐based approaches can also help compare the effects of different groups with similar functional roles (e.g., mammals versus birds) on the network structure and ecosystem functions. In practice, the functional interaction niche of a species can be quantified using one or more functional traits of its interaction partners. That is, the functional interaction niche of a species is the functional variation of its partners, which can be measured with a variety of metrics, such as trait range, trait variation, functional richness, functional evenness, or functional dispersion (Dehling et al. 2021; Junker et al. 2013; Phillips et al. 2020). These metrics exist in one‐ to *n*‐dimensional trait spaces and can be based on presence‐absence or interaction frequency data (Dehling et al. 2021; Junker et al. 2013). The properties of a species' functional interaction niche indicate in an ecologically relevant way the breadth of biotic conditions that the species can tolerate.

### 2.3 Fundamental Versus Realised Interaction Niches

It is not a trivial choice from which network the functional interaction niche of a species is quantified because the spatiotemporal scale affects the detection of interactions (Galiana et al. 2018). Moreover, the interactions in the network can be predicted using empirically observed interactions with various modelling approaches (Fricke et al. 2022). We consider two alternatives to quantify functional interaction niche properties: local empirically observed networks and a regional scale predicted metanetwork (hereafter, metanetwork). Using local empirical networks, we can quantify the realised functional interaction niches of all species present in that given network, thus accounting for the (co‐)occurrences of species inherently. Analogous to the environmental niche, the realised interaction niche is the set of interaction partners associated with the focal species (Dehling and Stouffer 2018).

A metanetwork covers predicted interactions between all species pairs in the region of interest. Using this metanetwork, we can quantify the fundamental functional interaction niches of all species present regionally, disregarding the (co‐)occurrences of species. Traditionally, the fundamental niche of a species is considered to be the entire set of abiotic environmental conditions it can tolerate. Similarly, the fundamental interaction niche is the complete set of partners with which a species can match functionally (irrespective of whether they have been observed interacting in local networks), allowing an interaction to be realised if the species pair co‐occurs. As we focus on fundamental interaction niches, our conceptual framework always operates in this ‘potential’ space within the metanetwork, covering only the feasible or functionally possible interactions. Thus, we do not account for the additional abiotic and biotic filters (e.g., within‐guild competition and abundance effects) that mediate turning feasible interactions into realised interactions in local networks.

Focusing on the fundamental interaction niche allows us to disregard the role of co‐occurrences in shaping the interactions between species. It also allows us to separate trait‐based mechanisms underlying pairwise interaction links from the patterns created by other mechanisms, such as environmental filtering. A metanetwork can be predicted based on traits as a small number of species traits govern the overall structure of interaction networks (Eklöf et al. 2013; Pires et al. 2011), and predicting interactions with statistical or rule‐based models is a common practice for various systems (Caron et al. 2024; Fricke and Svenning 2020; Pires 2017). We consider the fundamental interaction niches to be a more relevant aspect of interaction niches than the realised interaction niches in the context of future predictions. This is because it is not enough to know which species interact under the current conditions to discover which species could interact when community compositions are reshuffled under global change—a scenario that the fundamental interaction niches allow to unfold.

### 2.4 From Interaction Niche to Rewiring Potential

**FIGURE 2:**
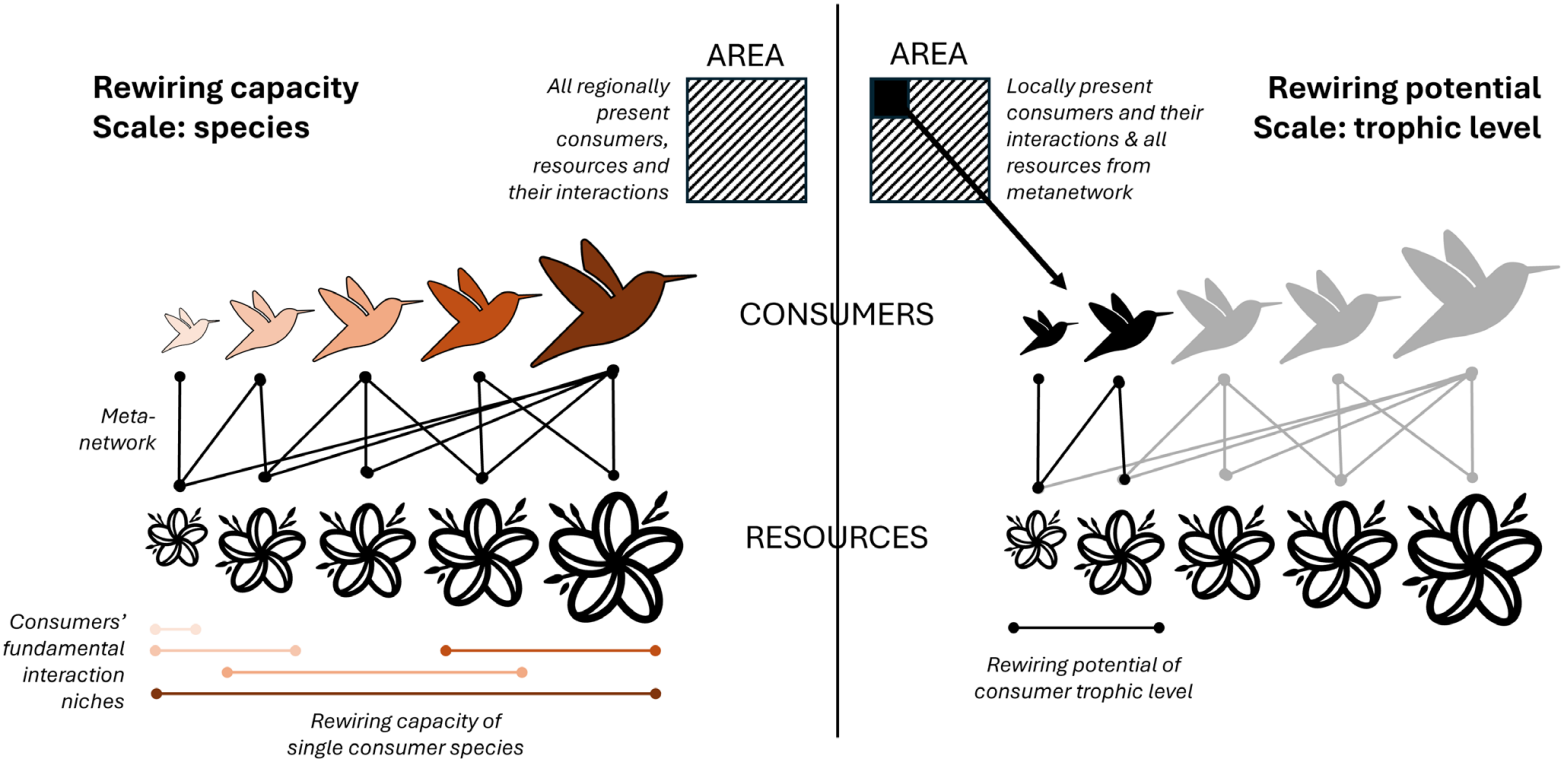
Schematic illustration of ‘rewiring capacity’ and ‘rewiring potential’ concepts derived from species functional interaction niches. On the left, the simplified resource‐consumer metanetwork (bird = consumer species, flower = resource species, black line = pairwise interaction) shows how rewiring capacity is quantified for single consumer species from their functional interaction niches (horizontal lines where colour corresponds to a consumer species). Rewiring capacity equals the fundamental interaction niche of the species based on the interaction partners' traits (flower sizes). On the right, the metanetwork is pruned to the locally present consumers and their interactions (black bird symbols and lines), including all their predicted partners from the metanetwork. Rewiring potential (horizontal black line) equals the total functional space (sizes of the two smallest flowers) covered by the interaction partners of the locally present consumers.

The functional interaction niche concept can be used to disentangle the resilience of pairwise interaction links and trophic levels against changing environmental conditions. It can be extended to quantify rewiring potential at the scales of single species and trophic levels (Figure 2); this concept of rewiring potential relates to rewiring pathways #1 (loss of interactions) and #2 (emergence of interactions) illustrated in Figure 1. However, we note that rewiring does not necessarily lead to positive outcomes for network resilience, especially for new species establishing in the network. For example, honeybee ( *Apis mellifera* ) could be viewed as a problematic invasive species outcompeting native species or as a functional replacement for the otherwise missing species providing plants with pollination (Valido et al. 2019).

At the species scale, the fundamental functional interaction niche size within the regional metanetwork can be directly used to derive the species ‘rewiring capacity’. At the trophic level scales, the ‘rewiring potential’ can be defined indirectly from the species' interaction niches by pruning the metanetwork to a local species community. These resulting pruned local networks represent feasible interactions that the locally occurring species are predicted to have, given their functional traits. Then, for all species within that community at the target trophic level simultaneously, the functional space covered by their interaction partners is quantified based on their pairwise interaction links with all species at the other trophic level in the metanetwork. Similarly to quantifying the interaction niche, rewiring potential can be measured with a variety of metrics, such as trait range, trait variation, functional richness, functional evenness, or functional dispersion (Dehling et al. 2021; Junker et al. 2013; Phillips et al. 2020). These metrics exist in one‐ to *n*‐dimensional trait spaces and can be based on presence‐absence or interaction frequency data (Dehling et al. 2021; Junker et al. 2013). Thus, each local network will have a number of rewiring capacity estimates equal to the number of species present and two trophic level‐specific rewiring potential estimates. The rewiring potential at the scale of a trophic level is based on a local network pruned to the species at the target trophic level present there. Noteworthy, the rewiring potential quantification will always be based on the metanetwork predicted based on interactions derived from trait matching. This means that the rewiring potential accounts for the future potential since pruning depends on whether the current or future community composition is used to compile local trophic levels. The higher the rewiring capacity or potential, the better a species or a network could withstand environmental changes (Figure 2).

### 2.5 Uses of Rewiring Potential

Our conceptual framework of rewiring potential at both scales can be applied to bipartite and multitrophic networks as long as two trophic levels of resources and consumers are considered at a time. In addition, the framework can be applied to both mutualistic and antagonistic (e.g., predator–prey and herbivore‐plant) interaction types. However, interaction resilience and rewiring potential only make sense from the perspective of those trophic levels that benefit from the pairwise interaction of interest (i.e., top–down). For example, a predator species benefits while a prey species does not benefit from the antagonistic predator–prey interaction occurring between the species. Therefore, it can be assumed that only a predator species will have an ecologically relevant rewiring capacity under changing environmental conditions, related to the functional interaction niche size of its prey species' body sizes. On the other hand, the rewiring capacity of a prey species will not determine its response to changing environmental conditions as the prey species should always benefit from the absence of its predators. This is because the evolutionary drivers are different for predator and prey species (Brodie and Brodie 1999; Zu et al. 2016). For mutualistic plant‐pollinator interactions, it can be assumed that species at both trophic levels benefit from the interaction, making the rewiring potential relevant for both trophic levels under changing environmental conditions.

### 2.6 Required Data

Quantifying rewiring potential and predicting network structure into the future requires extensive data on species' traits and pairwise interactions. Our conceptual framework can be applied to empirical datasets by leveraging the current broad availability of trait and interaction data (Bello et al. 2017; Enquist et al. 2016; Faurby et al. 2018; Tobias et al. 2022; Wilman et al. 2014) and the novel methodologies for network predictions based on traits and machine learning (Fricke et al. 2022). To apply the conceptual framework and quantify the rewiring capacity and potential, ecologically relevant traits for the interaction type in question and with a balance for all species at both trophic levels are needed to predict the metanetwork. This is because the number and choice of traits influence the measured properties of the *n*‐dimensional trait space used to quantify rewiring capacity and potential. The choice of traits should reflect the realisation of the interaction in natural conditions. For example, in predator–prey interactions, functional traits beyond morphological characteristics, such as related to behaviour, are crucial to include to encapsulate the determinants of which prey species a predator species can consume. Despite the increasing availability of trait data, gaps remain, especially when using large network datasets across broad geographical gradients. These gaps can be filled using (phylogenetic) imputation methods (Swenson 2014); however, these should be carefully explored to ensure that uncertainty in the imputation is appropriately accounted for in the subsequent rewiring capacity and potential calculations. In addition to trait data, observation data of presences and absences of pairwise interactions are needed to predict the metanetwork. Therefore, data stemming from complete surveys of local empirical networks are preferred. For the quantification of rewiring potential from the pruned metanetwork, local occurrence data of species at both trophic levels are also needed, for instance, from range maps (e.g., Enquist et al. 2016; Faurby et al. 2018).

## 3 Case Study on Plant‐Hummingbird Pollination Networks

### 3.1 Questions

We illustrate the application of the conceptual framework for mutualistic flower visitation (hereafter, pollination) networks of flowering plants and hummingbirds in the Americas. In this case study, we ask: (1) What is the rewiring capacity of plant versus hummingbird species, (2) What is the macroecological pattern of rewiring potential at the plant and hummingbird trophic levels, and (3) Is the rewiring potential of local networks related to species richness? The case study illustrates how to shed light on the patterns in functional resilience at species and trophic level scales, which can be used to propose hypotheses about the underlying mechanisms to be tested in the future. We describe the key information of interaction, trait, and range data filtering and the statistical analyses here and include the detailed version in the extended methods in Appendix S1.

### 3.2 Data

We obtained data from 79 complete bipartite plant‐hummingbird networks from Dalsgaard et al. (2021) with 1,002 plant species, 172 hummingbird species, and 4,155 observed pairwise interactions in the Americas. In addition, 10,814 absences of pairwise interactions were inferred from the complete networks if an interaction was not observed between a pair of plant and hummingbird species that occurred in a study site. We transformed the number of observed flower visits to binary presences and absences of interactions because of two reasons. Firstly, we assume that if an interaction has been observed, it is functionally feasible, and a low interaction frequency would not necessarily mean that such interaction is less likely in the metanetwork and fundamental interaction niche contexts. Other factors, such as competition, may limit the local interaction frequency, but the limitation may be removed in case community composition gets reshuffled (Dalsgaard and Temeles 2024). Secondly, as our dataset includes observed networks from a broad geographical region and from a wide range of studies with slightly different methods, we consider the use of the binary data to be more robust against possible sampling‐effort induced biases in the interaction frequencies. To extend beyond the observed hummingbird species in the 79 networks, we included 146 additional hummingbird species that consume nectar as their main food source (≥ 90% in EltonTraits; Wilman et al. 2014) and are included in the bird checklist of South, Central or North America (Lepage 2023).

We obtained data on the following plant species' traits: corolla length, floral colour following the pollination syndrome concept, nectar concentration, maximum height of the plant, mean seed length, and maximum seed mass (Vollstädt et al. 2025; Weigelt et al. 2019; Appendix S1, Table S1a). We use this broad array of plant traits to improve the ecological relevance and the capacity to predict interactions accurately. For those plant species with missing species‐level trait data, we imputed traits using an ancestral state reconstruction approach. We constructed a phylogeny by grafting missing species to Smith and Brown (2018) phylogeny using V.Phylomaker (Jin and Qian 2019). We obtained data on the following hummingbird species' traits: beak length, depth and curvature, tarsus length, hand‐wing index, tail length, body mass, and primary habitat association (Dalsgaard et al. 2021; Tobias et al. 2022; Appendix S1, Table S1b). We obtained plant species range maps from the BIEN database (Enquist et al. 2016) or range estimates based on GBIF observations (Appendix S1, Table S2). We obtained hummingbird ranges for both the species in the original networks and the additional species from BirdLife International (2022).

### 3.3 Fitting the Interaction Model

We used boosted regression tree methods to model the probability of interaction with the R package ‘gbm’ (Ridgeway et al. 2024). In the model, the response variable was the binary observed pairwise interaction, and the predictor variables were the species‐specific values of the plant and hummingbird traits (Appendix S1, Table S1). In addition, we included hummingbird clade (McGuire et al. 2014) and plant family as predictor variables to account for potential missing traits of which the taxonomic levels can be a proxy. We used the same tuning parameters as Fricke et al. (2022) and a 10‐fold cross‐validation to assess predictive performance on the randomly withheld tenth of the data (Elith et al. 2008). Following Fricke et al. (2022), we evaluated model fit using the area under the curve (AUC), Cohen's Kappa, and accuracy. In addition, we evaluated model fit using PR‐AUC and MCC (Poisot 2023), using R packages ‘yardstick’ and ‘mltools’ (Gorman 2018; Kuhn et al. 2024).

### 3.4 Predicting Interaction Probabilities

We used the fitted boosted regression model to predict the metanetwork, i.e., the feasible pairwise interaction probabilities of all possible flowering plant‐hummingbird species pairs. For this, we included all hummingbird species in the Americas. We predicted the interaction probabilities using the same traits and taxonomic groupings as in the model‐fitting step. This resulted in a probabilistic pollination metanetwork of 318,636 species pairs with predicted interaction probabilities. We then pruned the metanetwork to include only those interactions with a probability higher than 0.4. We set this threshold based on visual inspection of the inflection point from the variation in predicted probabilities against known observed/not‐observed values of the species' pairs that were present in the original network data (Appendix S1, Figure S1).

### 3.5 Quantifying Functional Interaction Niches

We used the pruned probabilistic metanetwork to quantify fundamental functional interaction niches for all 1,002 plant and 318 hummingbird species. We quantified these interaction niches using the *n*‐dimensional trait space volume for each species using the traits of its predicted interaction partners (Dehling et al. 2021; Junker et al. 2013). From this trait space, we quantified functional richness using R package ‘FD’ (Laliberté et al. 2014). Functional richness for multiple traits represents the amount of functional space filled by the community and is calculated as the volume of the *n*‐dimensional convex hull (Villéger et al. 2008). For example, for a hummingbird species with 15 predicted plant partners, the interaction niche was quantified as the functional richness of the 15 plant species. We used beak length, beak curvature, hand‐wing index, body mass, and tail length to calculate plant species' interaction niches, and floral corolla length, maximum height of the plant, nectar concentration, and floral colour to calculate hummingbird species' interaction niches.

### 3.6 Quantifying Rewiring Capacity and Rewiring Potential

The interaction niche size of a species represents the potential that the species has to find a functionally matching interaction partner under changing environmental conditions. Thus, the interaction niche size directly represents the rewiring capacity of a species. To quantify the spatially explicit rewiring potential for plant and hummingbird trophic levels, we collated the plant and hummingbird species' occurrences from range maps in 0.5 × 0.5° grid cells across the Americas. Then, for each trophic level separately, we quantified the functional richness of the interaction partners of all species within the trophic level. For example, for a plant community of 100 species present in a grid cell predicted to interact with 20 hummingbird species, we calculated the functional richness of the 20 hummingbird species using the same traits when quantifying interaction niches of single species. Thus, the total interaction niche size of all species present in the local species community represents the rewiring potential of a trophic level (Figure 2).

### 3.7 Disentangling Species Richness Effects on Rewiring Potential

To explore what kind of local networks have high or low rewiring potential, we assessed the relationships between trophic level‐specific rewiring potential and trophic level‐specific species richness using generalised additive models (GAMs) for each trophic level separately. We used the functional rewiring potential as the response variable. We included the species richness within the focal trophic level with maximum smoothing degrees of freedom set to *k* = 10 as the main predictor and the interaction of the grid cell centroid coordinates (~s(longitude, latitude)) with maximum smoothing degrees of freedom set to default as the additional predictor variable to control for spatial effects in the models. We used R package ‘mgcv’ to fit the models (Wood 2011). We then assessed the spatial variation in the disentangled effects using R package ‘biscale’ (Prener et al. 2022).

### 3.8 Results

**FIGURE 3:**
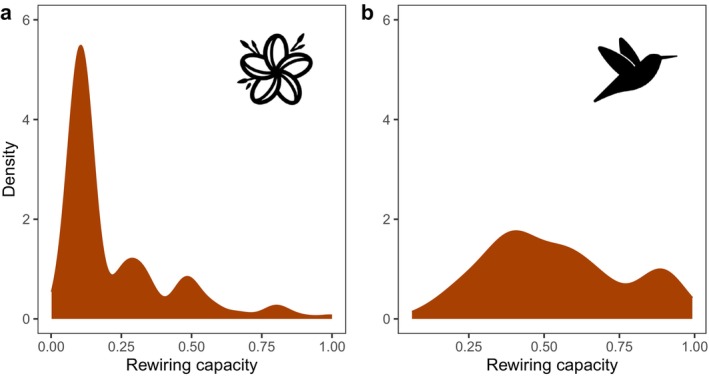
Density distribution of rewiring capacity among plant (panel a) and hummingbird (panel b) species. The *x*‐axis describes the rewiring capacity measured as the functional richness (FRic) of the interaction partners of each species based on functional traits, standardised within trophic level (0 = low functional richness, 1 = high functional richness).

**FIGURE 4:**
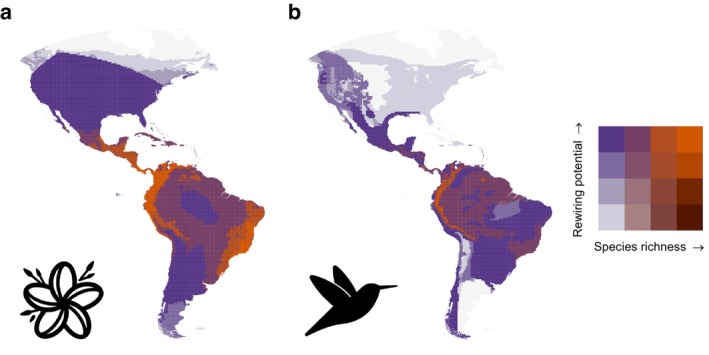
Spatial variation in rewiring potential and species richness across the Americas for plant (panel a) and hummingbird (panel b) trophic levels. The compositions of trophic levels are compiled from contemporary range maps within 0.5 × 0.5° grid cells. The orange colours (upper‐right corner of legend) indicate grid cells where both rewiring potential and species richness are high. In contrast, the bright blue colours (upper‐left corner of legend) indicate grid cells where species richness is low but rewiring potential is high. Light grey colour indicates areas with NA values (i.e., none of the plant or hummingbird ranges overlap with the grid cell).

Cross‐validated model performance was 0.784 (AUC), 0.361 (Kappa), 0.772 (accuracy), 0.834 (PR‐AUC), and 0.620 (MCC), indicating that the model fit was adequate. For (trait) variable importance, see Appendix S2, Table S1. When comparing the rewiring capacity of species, we found inequality between trophic levels such that, on average, plant species tended to have lower rewiring capacity than hummingbird species (Figure 3). We also found that the rewiring potential was high (> 0.8 in 67% of the grid cells for hummingbirds and 87% of the grid cells for plants) in most areas at both plant and hummingbird trophic levels (Figure 4). Modelling the effect of species richness on rewiring potential showed that species richness significantly increased rewiring potential even when controlling for the spatial coordinates (for full model output, see Appendix S2, Tables S2 and S3). In practice, the rewiring potential quickly saturated to maximum when richness increased beyond ~75 plant species and ~15 hummingbird species (Appendix S2, Figures S1 and S2).

## 4 Discussion

### 4.1 Summary

Here, we have outlined a conceptual framework for assessing species' and networks' rewiring potential and then used the framework to determine the flowering plant and hummingbird communities' rewiring potential in the Americas. Rewiring capacity represents the functional flexibility of a species and might only become empirically evident following a change in environmental conditions. Our framework contributes to a better understanding of the functional aspects of fundamental niches and rewiring potential. In that regard, our framework clearly distinguishes between realised interaction niches observed in empirical networks and rewiring capacities that encapsulate the fundamental functional interaction niches. These two do not have to be similar as rewiring capacity can be much greater than the realised interaction niches that we observe in nature.

Our work complements the earlier empirical research on the rewiring of ecological networks by considering interaction niches from fundamental and functional perspectives, allowing us to expand the spatial and taxonomic contexts beyond sampled localities and species. Manipulative and natural experiments have taught us that species may use a much larger array of interaction partners, fitting their functional potential if given the opportunity. For example, in a manipulation experiment, extensive pairwise interaction rewiring has been achieved with minimal changes in key ecological features in a lake ecosystem (Jonsson et al. 2005). That is, removing three planktivorous fish species and adding one piscivorous fish species substantially shuffled the lake's community structure. Still, species richness, body size distribution, and trophic level‐specific abundances did not change. Applying our framework to assess rewiring potential in such a lake ecosystem could provide information on the functional mechanisms contributing to the maintenance of key ecological features in changing environmental conditions. Likewise, hurricanes can be seen as natural experiments, which have taught us that surprising pollinators may take over the pollination niche when the coadapted pollinators disappear or are in low numbers after hurricanes (Dalsgaard and Temeles 2024). For instance, carpenter bees started pollinating otherwise bat‐adapted cacti (Rivera‐Marchand and Ackerman 2006), and an array of smaller hummingbirds and passerine birds started visiting and pollinating nectar‐rich plants when the largest coadapted hummingbird species strongly declined after hurricanes (Schrøder et al. 2024).

### 4.2 Case Study

In the case study, we illustrate the applicability of the conceptual framework and find three key ecological results related to the rewiring potential of plant–hummingbird pollination networks in the Americas.

Firstly, we observed an imbalance in the rewiring capacities between plant and hummingbird trophic levels, such that the hummingbird species tend to have a higher rewiring capacity than the plant species. This aligns with earlier studies that have used approaches based on species identities and found that plants and pollinators have different degrees of specialisation within networks. On average, plants tend to be more specialised than pollinators (Blüthgen et al. 2006). The causes of the differences between trophic levels are likely the differential abiotic and biotic filters at the two trophic levels. Simply put, there are fewer hummingbird species to “choose from” for the plants. However, we might also not be capturing the complete pollination process as some plants may also be visited by insects and other vertebrate pollinators (Albrecht et al. 2018; Dalsgaard et al. 2009; Iamara‐Nogueira et al. 2022), which would broaden the interaction niches of those plant species that appear to have narrow interaction niches. Although it is challenging to collectively consider the functional spaces defined by different taxonomic groups, in the future, the rewiring potential of plants should be assessed by including all possible pollinator taxa. We also note that many plants can self‐pollinate, meaning their reproduction may not fully depend on the hummingbird partners. We find larger fundamental interaction niches of hummingbird species compared to earlier studies on realised interaction niches (measured as specialisation using species identities; Dalsgaard et al. 2021), probably because we use a trait‐based approach. That is, based on traits, hummingbird species can interact with a large proportion of flowering plants, but their realised occurrences do not allow them to interact with all possible plant partners, likely due to competitive exclusion (Schrøder et al. 2024). It is possible that speciation and biotic (competitive exclusion) and abiotic (environmental) filtering are stronger processes determining the realised functional interaction niches of hummingbirds than the trait‐based fundamental interaction niche sizes predicted by traits alone. The observed differences in rewiring capacities between plants and hummingbirds have possible future ramifications for the pollination ecosystem function. It can be assumed that sessile plants shift their ranges slower in response to climate change than mobile hummingbird species, creating novel co‐occurrences of species pairs at these two trophic levels (Sonne et al. 2022). Under those novel conditions, the rewiring capacity imbalance will mean that an average hummingbird would relatively easily find resources from the selection of plant species available in the newly colonised areas. In contrast, pollination of an average plant species largely depends on the sample of hummingbird species existing and colonising the area. In other words, at first glance, hummingbirds should be less affected by global change than their nectar‐food plants.

Secondly, we generally observed a high rewiring potential when quantified for plant and hummingbird trophic levels in local networks across the Americas (here, delineated within grid cells). Rewiring potential was particularly high in the Andes for both plant and hummingbird trophic levels, indicating networks' resilience against future environmental changes. Similar resilience was observed by Sonne et al. (2022), who predicted few climate‐driven extinctions and coextinctions and high colonisation potential under the changing climate for the Andean pollination networks. In contrast, the rewiring potential between trophic levels varied most strongly in North America (Figure 4), where plants tended to have very high but hummingbirds had very low rewiring potential. This means that plant species currently occurring in the region could establish interactions with a large set of functionally different hummingbird species if they were to colonise the region. Conversely, hummingbird species presently occurring in the region could only establish interactions with a limited set of functionally rather similar plant species if new plant species were to colonise the region. Although the empirical data behind the predictions are limited in North America, our predictive framework provides a unique opportunity to assess networks' resilience using comprehensive data on plant and hummingbird traits. In general, the cause for a high rewiring potential of a trophic level within a local network is the presence of any number of species with broad functional interaction niches, i.e., functional generalists. Functional generalists are key contributors to the network‐level rewiring potential and networks' ability to maintain ecosystem functions. Along these lines, in the future, if functional specialists go extinct and functional generalists expand their ranges, interactions within pollination networks will likely be maintained despite the turnover in the species' identities.

Thirdly, we observed that species richness has an important mediating effect on the rewiring potential, such that high species richness always leads to high rewiring potential, but low species richness communities can have either high or low rewiring potential (Appendix S2, Figures S1 and S2). Species richness likely affects rewiring potential via redundancy in pairwise interactions predicted in the metanetwork. In a hypothetical situation, one hummingbird species visits a plant species in an ecosystem. In addition, there are two functionally suitable hummingbird species present (i.e., with redundant fundamental functional interaction niches), but they are currently competitively excluded from visiting the plant species by the first hummingbird species. If the first hummingbird species goes locally extinct, the competitive exclusion will be released, and the plant species will be pollinated by the two other hummingbird species instead. Thus, if many species are present locally (i.e., species richness is high), the likelihood that some overlap in their fundamental functional interaction niches increases, and so does the rewiring potential.

Despite the extensive data and analyses, our case study has caveats. Based on our model fit, we predicted interactions between species that have yet to be known to interact in the empirical dataset. Such absences of interaction in empirical data could be false negatives due to the non‐detection of the interaction, which could lead to decreased model performance. We also note that due to data gaps, fewer networks are observed in North America and the Amazon, which may lead to less informative estimates of rewiring potential in those regions. However, compared to the rest of the Americas, North America has few hummingbird‐adapted plants, and the flowering plant diversification rate has been higher in the Atlantic Forest and Andes biomes compared to the Amazon region (Dimitrov et al. 2023), indicating that a large part of the phylogenetic and potentially functional diversity of hummingbird‐pollinated plants is covered by the current dataset. We also recommend future studies to explore the determination of the threshold that divides the predicted interaction probabilities into predicted presences and absences (we used 0.4 as the threshold), applying existing methodology (Freeman and Moisen 2008; Jiménez‐Valverde and Lobo 2007; Liu et al. 2005, 2013) as the classification of the interaction probabilities into presence‐absences can affect the structure of the predicted metanetwork.

Our case study results have ecological and conservation relevance for hummingbird‐plant networks in the Americas because the rewiring capacity and rewiring potential represent quantitative assessments of the network resilience of the continents as a whole. Understanding what kind of networks (e.g., in terms of species richness) have high rewiring potential has broad applications. There is complementarity between rewiring potential and species richness to assess which species and pollination networks should be conserved to ensure the maintenance of pollination ecosystem function under future environmental changes. Depending on the conservation target, it may be most efficient and crucial to conserve pollination networks where both trophic levels have high rewiring potential but low species richness, such that they depend strongly on the presence of few species. For example, rewiring potential was high for both plants and hummingbirds in the southern half of South America, but simultaneously the number of species at both trophic levels remained low (Figure 4). In practice, conserving a few species could be planned more effectively and species‐specifically while ensuring the continuity of pollination function in the ecosystem. On the other hand, any area with low rewiring potential is highly vulnerable to the loss of any species and could therefore be targeted for conservation. Such areas occurred especially in North America, where the low number of hummingbird species coincides with low rewiring potential (Figure 4b). Habitat loss and climate change threaten pollination of flowering plant species via pollinators (Leimberger et al. 2022), showing that understanding which pollinators have the capacity to take over the roles of declining and disappearing species is useful for conservation.

### 4.3 Outstanding Questions and Applications From the Framework

Many fundamental questions remain in understanding network resilience variation (Table 1). We assume that rewiring is one of the processes determining how networks will respond to environmental disturbance. Our conceptual framework builds the first layer of understanding of how rewiring could contribute to network and ecosystem resilience. To confirm this assumption, future research should especially focus on establishing causal positive links between rewiring, network properties (e.g., robustness; Dunne et al. 2002), network resilience, and ecosystem stability, as such links remain logical but untested. For example, network resilience as considered in this paper is a testable component of ecosystem stability for future studies.

**TABLE 1:** Examples of outstanding questions in ecology that could be answered with the conceptual framework on rewiring capacity and rewiring potential.

Question	Potential application of the conceptual framework
Can other species assume the functional role of a lost species in the network and maintain ecosystem functioning?	Compare rewiring capacities and interaction niche sizes of the lost species and remaining species in the local empirical network
How high rewiring potential is needed to maintain an interaction‐dependent ecosystem function?	Define a minimum network or interaction property value, that is required to maintain the ecosystem function. Simulate networks with species of different rewiring capacities to obtain a threshold value
Is high contemporary rewiring potential associated with high stability in past network structure or species/phylogenetic diversity?	Quantify rewiring potential in local empirical networks at both trophic levels and correlate the measure to past network property and species richness or phylogenetic diversity index variation
Which species should be reintroduced to restore an ecosystem function of interest?	Quantify the rewiring capacities of candidate species. Compare the functional niche available of the interaction partners at the trophic level that are still present in the area with the interaction niches of the reintroduction candidate species. Identify the species with the most overlap
How likely is a non‐native species to establish or replace pairwise interaction links in a network?	Quantify rewiring capacity in the native range. Compare the interaction niches available among the interaction partners at the trophic level that are present in the area with the interaction niche of the non‐native species in its native range. Quantify proportional overlap
Which species could be introduced to an ecosystem to restore a lost functional role, such as seed disperser or herbivore, in a rewilding program?	Define the functional role to be filled (e.g., targeting specific resource plants). Select a species with a sufficient rewiring capacity and a suitable interaction niche in its native range
Which networks should be prioritised for conservation to ensure maintenance of ecosystem function under changing environmental conditions?	Quantify rewiring potential in local empirical networks. Prioritise networks that have high rewiring potential

The concepts of rewiring capacity and potential can be applied in conservation prioritisation, rewilding, and invasion ecology. In conservation prioritisation, species could be selected for reintroduction and rewilding efforts based on their rewiring capacity. Their functional interaction niche properties could be used to match the functional gaps in the ecosystems needing restoration (Pires 2017). In invasion ecology, the invasion risk of a species could be assessed with the rewiring capacity (Vizentin‐Bugoni et al. 2020), such that the higher the capacity, the higher the risk that the species will be able to establish in a new area. The benefit of the approach is that a species' risk can be assessed prior to invasion without data on known interactions with the native species in the area yet‐to‐be‐colonised.

Rewiring capacity and potential could also complement other relevant global change response measures, such that the low rewiring capacity of a species could operate in interaction or indirectly with other aspects of vulnerability to global change drivers (Dawson et al. 2011). For example, in animals, vulnerability to climate change and specialisation in resource use are often linked (Schleuning et al. 2016). This likely stems from the fact that range size and generalism are positively associated (Hurtado et al. 2024), and species with large ranges probably have broader climatic tolerances (Alzate and Onstein 2022; Lancaster 2022). In the applied ecology context, our framework contributes to guiding conservation that incorporates higher organisational levels of biodiversity. In general, there is a need to expand conservation beyond species and habitats to protect ecosystem functions. For this, networks are critical, and our rewiring potential metric provides a way to identify the areas where networks will resist the negative effects of species composition changes. For example, if the rewiring potential is quantified from multiple interaction types in a region, it can guide where to find and protect networks vulnerable to global change pressures, i.e., the ones with low rewiring potential. Similar attempts have been made using a non‐functional approach to estimate food web uniqueness in Europe (Gaüzère et al. 2023).

These outstanding questions are limited to questions on topological rewiring (i.e., loss and emergence of pairwise interactions; Figure 1), because our conceptual framework in its current form is best suited to answer ‘where’ a new species could fit in the network rather than how a new species would alter the strength of existing interactions in the network. In the future, the conceptual framework could be extended to cover interaction strength rewiring, which could be possible by modelling and predicting interaction strengths and pruning the metanetwork to local network using local abundances rather than occurrence data.

## 5 Conclusions

Our conceptual and methodological framework represents an advance in predicting the resilience of ecological networks globally, including regions where detailed interaction data are lacking. By leveraging data on species' interactions, traits, and occurrences, our framework quantifies ‘rewiring capacity’ and ‘rewiring potential’ and forecasts how ecological networks reorganise in response to environmental changes. This capability is crucial for understanding and managing biodiversity under global change, as it provides a robust tool for mapping the adaptive responses of ecological communities and ecosystems even in data‐poor areas. Our framework fundamentally shifts the paradigm from reactive to predictive ecology, equipping scientists and conservationists with the means to anticipate the impacts of global change on species interactions and ecosystem functions worldwide as well as guiding targeted actions to preserve and enhance network resilience across diverse biomes. Our plant‐hummingbird pollination case study results suggest a generally high resilience in pollination ecosystem function, taking a major step forward by showing that species are much more flexible functionally (measured with the fundamental functional interaction niches) than could be inferred from their realised interactions today. This conclusion generalises earlier smaller‐scale findings showing that species are flexible in their interaction partner matching when “pushed” by extreme environmental conditions. Our case study findings reveal that functional interaction niches and our proposed rewiring metrics have great potential as a tool for developing more mechanistic approaches to understand the impact of global change on ecological communities across interaction types and biomes. Understanding the resilience of species and networks can help prioritise efforts to conserve remaining biodiversity, select most useful species for rewilding of ecosystem functions, and identify which species pose the largest threats via biological invasions.

## Author Contributions

E.‐L.M. and A.O. initiated the study, B.D. provided data and insight for the case study, E.‐L.M. analysed the data and wrote the first draft of the manuscript, and all authors contributed to the design of the study and to the later versions of the manuscript.

### Peer Review

The peer review history for this article is available at https://www.webofscience.com/api/gateway/wos/peer‐review/10.1111/ele.70146.

## Supporting information


Data S1.

## Data Availability

Raw data on interaction, trait, range, and phylogenetic relationships are available via the cited references. Filtered and formatted data and code for the case study are available on Dryad: https://doi.org/10.5061/dryad.h9w0vt4sq.
