# Supplementary material for: Fundamental Interaction Niches: Towards a Functional Understanding of Ecological Networks' Resilience
Source: Ecol Lett. 2025 May 30;28(6):e70146. doi: 10.1111/ele.70146 (PMC12123259; doi:10.1111/ele.70146)
Supplement: Supplementary file 1 — Data S1. [file ELE-28-0-s001.docx]

**Marjakangas, Dalsgaard & Ordonez**

**Supplementary material of two appendices to paper “Fundamental interaction niches: towards a functional understanding of ecological networks’ resilience”**

**Appendix S1. Extended methods for the case study.**

Data

*Flowering plant-hummingbird pollination networks.* We used data of complete bipartite plant-hummingbird flower visitation (hereafter, pollination) networks across South, Central and North America (Dalsgaard *et al.* 2021). Interactions were measured as the number of observed flower visits in which the hummingbird extracted nectar while touching the reproductive structures. The interaction data have been collected by observing full communities within a habitat type. We excluded local networks with fewer than five plant species or three hummingbird species. In addition, we excluded interactions if a plant or hummingbird individual was not identified to species level. After filtering the dataset, we included 79 complete networks with 1,002 plant species, 172 hummingbird species, and 4,155 observed pairwise interactions. In addition, 10,814 absences of pairwise interactions were inferred from the complete networks if an interaction was not observed between a pair of plant and hummingbird species that occurred in the study site. We transformed the number of observed flower visits to binary presences and absences of interactions to allow fitting the probabilistic model.

*Traits.* We obtained data of the following plant species’ traits: corolla length, pollination syndrome, nectar concentration, maximum height, mean seed length, and maximum seed mass (Table S1a). We use this broad array of plant traits to increase model performance. For those plant species that lacked species-level trait data, we imputed trait values using the phylogeny. We constructed a phylogeny using V.Phylomaker (Jin & Qian 2019) with Smith phylogeny as a backbone (Smith & Brown 2018). Then, we imputed the trait values based on ten runs using R package ‘Rphylopars’ (Goolsby *et al.* 2024) and averaging the imputed trait values across the ten runs. We obtained data of the following hummingbird species’ traits: beak length, depth and curvature, tarsus length, hand-wing index, tail length, mass, and habitat (Table S1b). There were no missing values for hummingbird traits, except for *Chlorostilbon olivaresi*, for which we quantified beak length and curvature as genus-level means.

**Table S1.** Description and sources of plant and hummingbird traits included in the analyses.

| 1. **Plants** | | | |
| --- | --- | --- | --- |
| **Trait** | **Description** | **Number of missing values** | **Source** |
| Corolla length | Length of corolla in millimeters | 210 | Vollstädt et al. 2025 |
| Floral color | Floral color following the pollination syndrome concept. Approximated as categorical flower color that indicates pollination syndrome. Continuous average based on multiple observations per species ranging between 1 and 3. 1 = short-wavelength color of blue and white and usually insect-pollinated, 3 = long-wavelength color of red and orange and usually bird-pollinated. | 76 | Vollstädt et al. 2025 |
| Nectar concentration | Nectar concentration in percentages | 450 | (Vollstädt et al. 2025 |
| Maximum height | Maximum plant height in meters | 742 | Weigelt et al. 2019 |
| Mean seed length | Mean seed length in millimeters | 977 | Weigelt et al. 2019 |
| Maximum seed mass | Maximum seed mass in grams | 893 | Weigelt et al. 2019 |
| 1. **Hummingbirds** | | | |
| **Trait** | **Description** | **Number of missing values** | **Reference** |
| Beak length | Length from the tip of the beak to the base of the skull | 0 | Tobias et al. 2022 |
| Beak depth | Depth of the beak at the anterior edge of the nostrils | 0 | Tobias et al. 2022 |
| Beak curvature | Mean bill curvature in degrees | 138 | Dalsgaard et al. 2021 |
| Tarsus length | Length of the tarsus from the posterior notch between tibia and tarsus, to the end of the last scale of acrotarsium (at the bend of the foot) | 0 | Tobias et al. 2022 |
| Hand-wing index | 100*DK/Lw, where DK is Kipp’s distance and Lw is wing length (i.e., Kipp’s distance corrected for wing size). Kipp's distance = length from the tip of the first secondary feather to the tip of the longest primary. | 0 | Tobias et al. 2022 |
| Tail length | Distance between the tip of the longest rectrix and the point at which the two central rectrices protrude from the skin, typically measured using a ruler inserted between the two central rectrices | 0 | Tobias et al. 2022 |
| Body mass | Body mass given as species average (both male and female) | 0 | Tobias et al. 2022 |
| Habitat | Main habitat associated with, levels: Desert (drylands and other open arid habitats, often sandy with very sparse vegetation);  Rock (rocky substrate typically with no/very little vegetation, including rocky outcrops, rocky coastlines, arid stony steppes, rocky mountaintops and mountain slopes);  Grassland (open dry to moist grass-dominated landscapes, at all elevations);  Shrubland (low stature bushy habitats, included thornscrub, thorny or arid savanna, caatinga, xerophytic shrubland and coastal scrub);  Woodland (medium stature tree-dominated habitats, including Acacia woodland, riparian woodlands, mangrove forests, forest edges, also more open parkland with scattered taller trees);  Forest (tall tree-dominated vegetation with more or less closed canopy, including palm forest);  Human modified (urban landscapes, intensive agriculture, gardens);  Wetland (wide range of freshwater aquatic habitats including lakes, marshes, swamps and reedbeds);  Riverine (rivers and streams at all elevations);  Coastal (intertidal zones within immediate vicinity of beaches, estuaries, brackish to salty marshes, including mudflats, lagoons, alkaline wetlands, coastal dunes and harbours);  Marine (pelagic, on sea near coasts, intertidal zone on beaches, pelagic sea cliffs, islets and islands) | 0 | Tobias et al. 2022 |

*Additional hummingbird species.* To extend beyond the observed hummingbird species in the 79 networks, we predicted interactions for 146 additional hummingbird species. We selected those hummingbird species that consume nectar as their main food source (>= 90% in EltonTraits; Wilman et al. 2014) and are included in the Avibase checklist of South, Central or North America (Lepage 2023). We selected the 90% nectar consumption threshold as it aligns with the nectar consumption of the hummingbird species observed in the original networks and thus prevents overpredictions. We excluded those species with taxonomic discrepancies that could not be solved with synonyms from the AVONET database (Tobias *et al.* 2022). We obtained the same traits for the additional hummingbird species as for the ones in the original dataset. There were no missing trait values, except for beak curvature for 138 species for which we calculated the genus-level means. The trait value ranges of the additional hummingbirds aligned with those of the observed hummingbird species, which justifies the inclusion of the additional species.

*Ranges.* We obtained plant species range maps from the BIEN database for 880 species (Enquist *et al.* 2016) using R package ‘BIEN’ (Maitner 2023). For the species lacking taxonomic matches in BIEN but including a taxonomic synonym in GBIF, we used the GBIF synonym to obtain the range map from BIEN. For the remaining 122 plant species lacking range maps in BIEN, we followed the BIEN range calculation protocol to estimate the ranges to ensure comparability across species (Merow 2017). First, we downloaded species’ observations from GBIF (Global Biodiversity Information Facility; see Table S2 for full list of data citations) and excluded doubtful observations using R Package CoordinateCleaner (Zizka *et al.* 2019). That is, we excluded observations in the ocean, in country centroids, botanical gardens, clear outliers if observation stemmed from iNaturalist, and observations at taxonomic levels other than species. Then, we obtained the range in one of three ways depending on the number of filtered GBIF observations: i) if N_observations_ > 5 we fitted a convex hull around the observations, ii) if N_observations_ <= 5 we made a bounding box around the minimum and maximum latitude and longitude coordinates, and iii) if if N_observations_ = 1 we made a 0.5 degree bounding box around the observation coordinates. No GBIF observations were available for eight of the 122 species. For those species, we built the ranges similarly based on the coordinates of the original networks where the plant species occurred (Table S3). We obtained hummingbird ranges for both the species in the original networks and the additional species (BirdLife International 2022). We used taxonomic synonyms following the taxonomic cross walk of AVONET (Tobias *et al.* 2022) when necessary.

**Table S2.** List GBIF data references.

GBIF.org (02 February 2024) GBIF Occurrence Download https://doi.org/10.15468/dl.k7ense

GBIF.org (02 February 2024) GBIF Occurrence Download https://doi.org/10.15468/dl.b26y8g

GBIF.org (02 February 2024) GBIF Occurrence Download https://doi.org/10.15468/dl.n4us7q

GBIF.org (02 February 2024) GBIF Occurrence Download https://doi.org/10.15468/dl.xvd2db

GBIF.org (02 February 2024) GBIF Occurrence Download https://doi.org/10.15468/dl.sh2m39

GBIF.org (02 February 2024) GBIF Occurrence Download https://doi.org/10.15468/dl.vkjtma

GBIF.org (02 February 2024) GBIF Occurrence Download https://doi.org/10.15468/dl.7htj86

GBIF.org (02 February 2024) GBIF Occurrence Download https://doi.org/10.15468/dl.refbyh

GBIF.org (02 February 2024) GBIF Occurrence Download https://doi.org/10.15468/dl.3n33np

GBIF.org (02 February 2024) GBIF Occurrence Download https://doi.org/10.15468/dl.phzjpp

GBIF.org (02 February 2024) GBIF Occurrence Download https://doi.org/10.15468/dl.rh988t

GBIF.org (02 February 2024) GBIF Occurrence Download https://doi.org/10.15468/dl.jnbawb

GBIF.org (02 February 2024) GBIF Occurrence Download https://doi.org/10.15468/dl.fesvb3

GBIF.org (02 February 2024) GBIF Occurrence Download https://doi.org/10.15468/dl.7bweyf

GBIF.org (02 February 2024) GBIF Occurrence Download https://doi.org/10.15468/dl.qm6pht

GBIF.org (02 February 2024) GBIF Occurrence Download https://doi.org/10.15468/dl.mk5cpz

GBIF.org (02 February 2024) GBIF Occurrence Download https://doi.org/10.15468/dl.2k9c5f

GBIF.org (02 February 2024) GBIF Occurrence Download https://doi.org/10.15468/dl.96anax

GBIF.org (02 February 2024) GBIF Occurrence Download https://doi.org/10.15468/dl.2c64dz

GBIF.org (02 February 2024) GBIF Occurrence Download https://doi.org/10.15468/dl.xgwpqv

GBIF.org (02 February 2024) GBIF Occurrence Download https://doi.org/10.15468/dl.u9t7c4

GBIF.org (02 February 2024) GBIF Occurrence Download https://doi.org/10.15468/dl.ed2wqu

GBIF.org (02 February 2024) GBIF Occurrence Download https://doi.org/10.15468/dl.subeny

GBIF.org (02 February 2024) GBIF Occurrence Download https://doi.org/10.15468/dl.6tkdmt

GBIF.org (02 February 2024) GBIF Occurrence Download https://doi.org/10.15468/dl.h7vnun

GBIF.org (02 February 2024) GBIF Occurrence Download https://doi.org/10.15468/dl.62d58v

GBIF.org (02 February 2024) GBIF Occurrence Download https://doi.org/10.15468/dl.w7rvsv

GBIF.org (02 February 2024) GBIF Occurrence Download https://doi.org/10.15468/dl.kxb8qp

GBIF.org (02 February 2024) GBIF Occurrence Download https://doi.org/10.15468/dl.krrs5y

GBIF.org (02 February 2024) GBIF Occurrence Download https://doi.org/10.15468/dl.cfhbja

GBIF.org (02 February 2024) GBIF Occurrence Download https://doi.org/10.15468/dl.jxb4d5

GBIF.org (02 February 2024) GBIF Occurrence Download https://doi.org/10.15468/dl.zawevb

GBIF.org (02 February 2024) GBIF Occurrence Download https://doi.org/10.15468/dl.bpsyn5

GBIF.org (02 February 2024) GBIF Occurrence Download https://doi.org/10.15468/dl.r3ugum

GBIF.org (02 February 2024) GBIF Occurrence Download https://doi.org/10.15468/dl.qaky82

GBIF.org (02 February 2024) GBIF Occurrence Download https://doi.org/10.15468/dl.nrgagd

GBIF.org (02 February 2024) GBIF Occurrence Download https://doi.org/10.15468/dl.rfd3a3

GBIF.org (02 February 2024) GBIF Occurrence Download https://doi.org/10.15468/dl.gfsgv5

GBIF.org (02 February 2024) GBIF Occurrence Download https://doi.org/10.15468/dl.9nvqmr

GBIF.org (02 February 2024) GBIF Occurrence Download https://doi.org/10.15468/dl.h9wheb

GBIF.org (02 February 2024) GBIF Occurrence Download https://doi.org/10.15468/dl.4xvjpr

GBIF.org (02 February 2024) GBIF Occurrence Download https://doi.org/10.15468/dl.s6errf

GBIF.org (02 February 2024) GBIF Occurrence Download https://doi.org/10.15468/dl.y76afp

GBIF.org (02 February 2024) GBIF Occurrence Download https://doi.org/10.15468/dl.fxdkc6

GBIF.org (02 February 2024) GBIF Occurrence Download https://doi.org/10.15468/dl.53wmjh

GBIF.org (02 February 2024) GBIF Occurrence Download https://doi.org/10.15468/dl.bj2pm2

GBIF.org (02 February 2024) GBIF Occurrence Download https://doi.org/10.15468/dl.fq9gex

GBIF.org (02 February 2024) GBIF Occurrence Download https://doi.org/10.15468/dl.7mpqfk

GBIF.org (02 February 2024) GBIF Occurrence Download https://doi.org/10.15468/dl.qfwfx9

GBIF.org (02 February 2024) GBIF Occurrence Download https://doi.org/10.15468/dl.jmmmsj

GBIF.org (02 February 2024) GBIF Occurrence Download https://doi.org/10.15468/dl.vguuwg

GBIF.org (02 February 2024) GBIF Occurrence Download https://doi.org/10.15468/dl.ujqbt5

GBIF.org (02 February 2024) GBIF Occurrence Download https://doi.org/10.15468/dl.q2q2v7

GBIF.org (02 February 2024) GBIF Occurrence Download https://doi.org/10.15468/dl.3h793w

GBIF.org (02 February 2024) GBIF Occurrence Download https://doi.org/10.15468/dl.d39uwe

GBIF.org (02 February 2024) GBIF Occurrence Download https://doi.org/10.15468/dl.ez7r22

GBIF.org (02 February 2024) GBIF Occurrence Download https://doi.org/10.15468/dl.5czsma

GBIF.org (02 February 2024) GBIF Occurrence Download https://doi.org/10.15468/dl.dxjunr

GBIF.org (02 February 2024) GBIF Occurrence Download https://doi.org/10.15468/dl.2qthcn

GBIF.org (02 February 2024) GBIF Occurrence Download https://doi.org/10.15468/dl.6zsuwd

GBIF.org (02 February 2024) GBIF Occurrence Download https://doi.org/10.15468/dl.mgj6r5

GBIF.org (02 February 2024) GBIF Occurrence Download https://doi.org/10.15468/dl.5x3ccz

GBIF.org (02 February 2024) GBIF Occurrence Download https://doi.org/10.15468/dl.9gjzgs

GBIF.org (02 February 2024) GBIF Occurrence Download https://doi.org/10.15468/dl.fnnh25

GBIF.org (02 February 2024) GBIF Occurrence Download https://doi.org/10.15468/dl.faqwzb

GBIF.org (02 February 2024) GBIF Occurrence Download https://doi.org/10.15468/dl.4aux87

GBIF.org (02 February 2024) GBIF Occurrence Download https://doi.org/10.15468/dl.bndtwq

GBIF.org (02 February 2024) GBIF Occurrence Download https://doi.org/10.15468/dl.9yaxgk

GBIF.org (02 February 2024) GBIF Occurrence Download https://doi.org/10.15468/dl.r74rst

GBIF.org (02 February 2024) GBIF Occurrence Download https://doi.org/10.15468/dl.j8jsmy

GBIF.org (02 February 2024) GBIF Occurrence Download https://doi.org/10.15468/dl.gfjdbg

GBIF.org (02 February 2024) GBIF Occurrence Download https://doi.org/10.15468/dl.3mc9d4

GBIF.org (02 February 2024) GBIF Occurrence Download https://doi.org/10.15468/dl.xjrj7e

GBIF.org (02 February 2024) GBIF Occurrence Download https://doi.org/10.15468/dl.ky8pzj

GBIF.org (02 February 2024) GBIF Occurrence Download https://doi.org/10.15468/dl.d59e64

GBIF.org (02 February 2024) GBIF Occurrence Download https://doi.org/10.15468/dl.a4b8md

GBIF.org (02 February 2024) GBIF Occurrence Download https://doi.org/10.15468/dl.yt2y8q

GBIF.org (02 February 2024) GBIF Occurrence Download https://doi.org/10.15468/dl.a9aqed

GBIF.org (02 February 2024) GBIF Occurrence Download https://doi.org/10.15468/dl.r29hf4

GBIF.org (02 February 2024) GBIF Occurrence Download https://doi.org/10.15468/dl.bg9rwf

GBIF.org (02 February 2024) GBIF Occurrence Download https://doi.org/10.15468/dl.fps3ce

GBIF.org (02 February 2024) GBIF Occurrence Download https://doi.org/10.15468/dl.8ugzf3

GBIF.org (02 February 2024) GBIF Occurrence Download https://doi.org/10.15468/dl.r6tvm6

GBIF.org (02 February 2024) GBIF Occurrence Download https://doi.org/10.15468/dl.nhgbxw

GBIF.org (02 February 2024) GBIF Occurrence Download https://doi.org/10.15468/dl.xc2bre

GBIF.org (02 February 2024) GBIF Occurrence Download https://doi.org/10.15468/dl.bzv3em

GBIF.org (02 February 2024) GBIF Occurrence Download https://doi.org/10.15468/dl.s64zph

GBIF.org (02 February 2024) GBIF Occurrence Download https://doi.org/10.15468/dl.uyf9q6

GBIF.org (02 February 2024) GBIF Occurrence Download https://doi.org/10.15468/dl.cv6jz4

GBIF.org (02 February 2024) GBIF Occurrence Download https://doi.org/10.15468/dl.nrv4a8

GBIF.org (02 February 2024) GBIF Occurrence Download https://doi.org/10.15468/dl.66u3jv

GBIF.org (02 February 2024) GBIF Occurrence Download https://doi.org/10.15468/dl.hyd2c8

GBIF.org (02 February 2024) GBIF Occurrence Download https://doi.org/10.15468/dl.7muyyf

GBIF.org (02 February 2024) GBIF Occurrence Download https://doi.org/10.15468/dl.4gxwvt

GBIF.org (02 February 2024) GBIF Occurrence Download https://doi.org/10.15468/dl.r3yq5w

GBIF.org (02 February 2024) GBIF Occurrence Download https://doi.org/10.15468/dl.tdkm3u

GBIF.org (02 February 2024) GBIF Occurrence Download https://doi.org/10.15468/dl.2hu6t2

GBIF.org (02 February 2024) GBIF Occurrence Download https://doi.org/10.15468/dl.qznrsc

GBIF.org (02 February 2024) GBIF Occurrence Download https://doi.org/10.15468/dl.rrrve3

GBIF.org (02 February 2024) GBIF Occurrence Download https://doi.org/10.15468/dl.xymy85

GBIF.org (02 February 2024) GBIF Occurrence Download https://doi.org/10.15468/dl.23dkju

GBIF.org (02 February 2024) GBIF Occurrence Download https://doi.org/10.15468/dl.pr9p7j

GBIF.org (02 February 2024) GBIF Occurrence Download https://doi.org/10.15468/dl.6xnjbt

GBIF.org (02 February 2024) GBIF Occurrence Download https://doi.org/10.15468/dl.x4wgre

GBIF.org (02 February 2024) GBIF Occurrence Download https://doi.org/10.15468/dl.hbqy87

GBIF.org (02 February 2024) GBIF Occurrence Download https://doi.org/10.15468/dl.cxvjp5

GBIF.org (02 February 2024) GBIF Occurrence Download https://doi.org/10.15468/dl.h6knpg

GBIF.org (02 February 2024) GBIF Occurrence Download https://doi.org/10.15468/dl.ctv92g

GBIF.org (02 February 2024) GBIF Occurrence Download https://doi.org/10.15468/dl.zgr2t6

GBIF.org (02 February 2024) GBIF Occurrence Download https://doi.org/10.15468/dl.xr4nby

GBIF.org (02 February 2024) GBIF Occurrence Download https://doi.org/10.15468/dl.7pbkwt

GBIF.org (02 February 2024) GBIF Occurrence Download https://doi.org/10.15468/dl.hc2jbx

GBIF.org (02 February 2024) GBIF Occurrence Download https://doi.org/10.15468/dl.mwcf9q

GBIF.org (02 February 2024) GBIF Occurrence Download https://doi.org/10.15468/dl.ynkcdg

GBIF.org (02 February 2024) GBIF Occurrence Download https://doi.org/10.15468/dl.gz5b43

GBIF.org (02 February 2024) GBIF Occurrence Download https://doi.org/10.15468/dl.gpcxes

GBIF.org (02 February 2024) GBIF Occurrence Download https://doi.org/10.15468/dl.m7r7zd

GBIF.org (02 February 2024) GBIF Occurrence Download https://doi.org/10.15468/dl.xeq224

GBIF.org (02 February 2024) GBIF Occurrence Download https://doi.org/10.15468/dl.39xgsh

GBIF.org (02 February 2024) GBIF Occurrence Download https://doi.org/10.15468/dl.r6egba

GBIF.org (02 February 2024) GBIF Occurrence Download https://doi.org/10.15468/dl.52htb4

GBIF.org (02 February 2024) GBIF Occurrence Download https://doi.org/10.15468/dl.ry2xp7

GBIF.org (02 February 2024) GBIF Occurrence Download https://doi.org/10.15468/dl.4gvwfr

GBIF.org (02 February 2024) GBIF Occurrence Download https://doi.org/10.15468/dl.kxwrt6

GBIF.org (02 February 2024) GBIF Occurrence Download https://doi.org/10.15468/dl.bxvgyq

GBIF.org (02 February 2024) GBIF Occurrence Download https://doi.org/10.15468/dl.gkjg97

GBIF.org (02 February 2024) GBIF Occurrence Download https://doi.org/10.15468/dl.acp9t5

GBIF.org (02 February 2024) GBIF Occurrence Download https://doi.org/10.15468/dl.9v2ge7

GBIF.org (02 February 2024) GBIF Occurrence Download https://doi.org/10.15468/dl.8j58u4

GBIF.org (02 February 2024) GBIF Occurrence Download https://doi.org/10.15468/dl.k4jg8p

GBIF.org (02 February 2024) GBIF Occurrence Download https://doi.org/10.15468/dl.rr9mf5

**Table S3.** List of species without GBIF observations.

*Bomarea polyneura*

*Canistrum terminalis*

*Centropogon isahellinus*

*Collandra petiolaris*

*Hippeastrum aviflorum*

*Lymania brachycaulis*

*Nidularium seidelii*

*Opuntia werneri*

Statistical methods

*Fitting the interaction model.* We used boosted regression tree methods to model the probability of interaction. This method allows studying the binomially distributed response variable of observed/not-observed pairwise interactions and, through cross validation, avoids overfitting of the multi-trait models. In the model, the predictor variables were the species-specific values of the plant and hummingbird traits mentioned above (Table S1). In addition, we included hummingbird clade (McGuire *et al.* 2014) and plant family as predictor variables. To implement these models, we used R package ‘gbm’ (Ridgeway 2024). We used the same set of tuning parameters as Fricke et al. (2022) and a 10-fold cross validation to assess predictive performance on the randomly withheld tenth of the data (Elith *et al.* 2008). To evaluate model fit for the model with binary response variables, we reported area under the curve (AUC), Cohen’s Kappa, and accuracy (i.e. outputting binary interaction predictions based on a cutoff of 0.5 predicted interaction probability).

*Predicting interaction probabilities.* We used the model fit to predict pairwise interaction probabilities to all possible flowering plant-hummingbird pairs, including those with additional hummingbird species. We did this by using the same set of traits and taxonomic groupings as in the model fitting step. This resulted in a probabilistic pollination network consisting of 318,636 species pairs with predicted interaction probabilities. We then pruned the metanetwork to include only those interactions with probability higher than 0.4. We set this threshold based on visual inspection of the inflection point from the variation in predicted probabilities against known observed/not-observed values of the pairs of species that were present in the original network data (Figure S1).


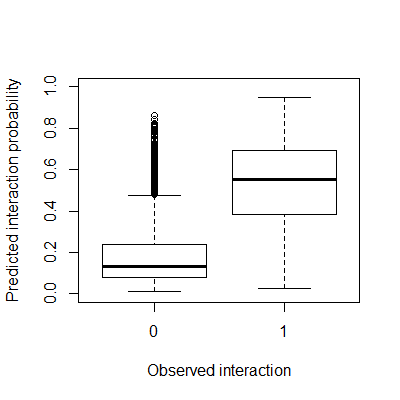


**Figure S1.** Differences in observed binary interactions and predicted interaction probabilities. N = 14,969 pairwise interactions occurring in the network dataset. The figure shows 1^st^, 2^nd^, and 3^rd^ quantiles as lines and outliers as points.

*Quantifying functional interaction niches and rewiring potential.* We used the pruned probabilistic metanetwork to quantify fundamental functional interaction niches for all 1002 plant and 318 hummingbird species. We quantified the fundamental functional interaction niche from the n-dimensional trait space volume for a focal species using the traits of its predicted interaction partners following earlier studies (Dehling *et al.* 2021; Junker *et al.* 2013). From this trait space, we quantified functional richness using R package ‘FD’ (Laliberté *et al.* 2014). Functional richness for multiple traits represents the amount of functional space filled by the community and is calculated as the volume of the n-dimensional convex hull (Villéger *et al.* 2008). We reduced the functional space dimension to three for computational efficiency. For example, for a hummingbird species with 15 predicted plant partners, the interaction niche was quantified as the functional richness of the 15 plant species. We used beak length, beak curvature, hand-wing index, body mass, and tail length to calculate plant species’ interaction niches and corolla length, maximum height, maximum seed length, mean seed mass, nectar concentration, and pollination syndrome to calculate hummingbird species’ interaction niches. In addition, we quantified the number of predicted interaction partners as this is often measured in other studies.

The fundamental functional interaction niche describes how functionally different partners can a focal species interact with, disregarding the realized co-occurrences of species. The interaction niche size represents the potential that a species has to find a functionally matching interaction partner under changing environmental conditions.

We also quantified the spatially explicit rewiring potential for local communities. We did this by collating the plant and hummingbird species’ occurrences from range maps in 0.5*0.5 degree grid cells across the Americas. Then, for each trophic level separately, we quantified the functional richness and functional evenness of the interaction partners of all species within the trophic level. For example, for a plant community of 100 species in a degree grid cell predicted to interact with 20 hummingbird species, we calculate the functional richness of the 20 hummingbird species using the same traits when quantifying interaction niches of single species.

*Disentangling species richness effects on rewiring potential.* To assess whether functional rewiring potential stems from the sheer number of species present in the local network, we assessed the relationships between trophic level-specific rewiring potential and trophic level-specific species richness. For this, we fitted generalized additive models for each trophic level separately. These models allow non-parametric relationships and thus high flexibility in the rewiring potential-species richness relationships compared to linear relationships. We used the functional rewiring potential as the response variable. We included the species richness within the focal trophic level with maximum smoothing degrees of freedom set to k = 10 as the main predictor and the interaction of the grid cell centroid coordinates (~s(longitude, latitude)) with maximum smoothing degrees of freedom set to default as the additional predictor variable to control for spatial effects in the models. We used R package ‘mgcv’ to fit the models (Wood 2011). We then assessed the spatial variation in the disentangled effects using R package ‘biscale’ (Prener *et al.* 2022).

**References**

BirdLife International. (2022). *Bird species distribution maps of the world*. Available at: http://datazone.birdlife.org/species/requestdis. Last accessed .

Dalsgaard, B., Maruyama, P.K., Sonne, J., Hansen, K., Zanata, T.B., Abrahamczyk, S., *et al.* (2021). The influence of biogeographical and evolutionary histories on morphological trait‐matching and resource specialization in mutualistic hummingbird–plant networks. *Funct Ecol*, 35, 1120–1133.

Dehling, D.M., Bender, I.M.A., Blendinger, P.G., Böhning‐Gaese, K., Muñoz, M.C., Neuschulz, E.L., *et al.* (2021). Specialists and generalists fulfil important and complementary functional roles in ecological processes. *Funct Ecol*, 35, 1810–1821.

Elith, J., Leathwick, J.R. & Hastie, T. (2008). A working guide to boosted regression trees. *Journal of Animal Ecology*, 77, 802–813.

Enquist, B.J., Condit, R., Peet, B., Schildhauer, M., Thiers, B. & BIEN working group. (2016). Cyberinfrastructure for an integrated botanical information network to investigate the ecological impacts of global climate change on plant biodiversity. *PeerJ Prepr*, 1–32.

Fricke, E.C., Ordonez, A., Rogers, H.S. & Svenning, J.-C. (2022). The effects of defaunation on plants’ capacity to track climate change. *Science (1979)*, 375, 210–214.

Goolsby, E., Bruggeman, J. & Ane, C. (2024). Rphylopars: Phylogenetic Comparative Tools for  Missing Data and Within-Species Variation.

Jin, Y. & Qian, H. (2019). V.PhyloMaker: an R package that can generate very large phylogenies for vascular plants. *Ecography*, 42, 1353–1359.

Junker, R.R., Blüthgen, N., Brehm, T., Binkenstein, J., Paulus, J., Martin Schaefer, H., *et al.* (2013). Specialization on traits as basis for the niche‐breadth of flower visitors and as structuring mechanism of ecological networks. *Funct Ecol*, 27, 329–341.

Laliberté, E., Legendre, P. & Shipley, B. (2014). FD: measuring functional diversity from multiple traits, and other tools for functional ecology. R package.

Lepage, D. (2023). *Avibase - The World Bird Database*. *https://avibase.bsc-eoc.org/checklist.jsp?region=index&lang=FI&list=clements*.

Maitner, B. (2023). BIEN: Tools for Accessing the Botanical Information and Ecology  Network Database.

McGuire, J.A., Witt, C.C., Remsen, J.V., Corl, A., Rabosky, D.L., Altshuler, D.L., *et al.* (2014). Molecular Phylogenetics and the Diversification of Hummingbirds. *Current Biology*, 24, 910–916.

Merow, C. (2017). *BIEN Range Methods Description*.

Prener, C., Grossenbacher, T. & Zehr, A. (2022). biscale: Tools and Palettes for Bivariate Thematic Mapping.

Ridgeway, G. (2024). gbm: Generalized Boosted Regression Models.

Smith, S.A. & Brown, J.W. (2018). Constructing a broadly inclusive seed plant phylogeny. *Am J Bot*, 105, 302–314.

Tobias, J.A., Sheard, C., Pigot, A.L., Devenish, A.J.M., Yang, J., Sayol, F., *et al.* (2022). AVONET: morphological, ecological and geographical data for all birds. *Ecol Lett*, 25, 581–597.

Villéger, S., Mason, N.W.H. & Mouillot, D. (2008). New multidimensional diversity indices for a multifaceted framework in functional ecology. *Ecology*, 89, 2290–2301.

Vollstädt, M.G.R., Jensen, R.D., Maruyama, P.K., Schleuning, M., Araújo-Hoffmann, F.P., Sazima, M., *et al.* (2025). The role of insularity: plants have few ornithophilous traits but are visited by morphologically more distinct hummingbirds in the Caribbean islands. *Funct Ecol*.

Weigelt, P., Koenig, C. & Kreft, H. (2019). GIFT - A Global Inventory of Floras and Traits for macroecology and biogeography. *J Biogeogr*.

Wilman, H., Belmaker, J., Simpson, J., de la Rosa, C., Rivadeneira, M.M. & Jetz, W. (2014). EltonTraits 1.0: Species-level foraging attributes of the world’s birds and mammals. *Ecology*, 95, 2027–2027.

Wood, S.N. (2011). Fast Stable Restricted Maximum Likelihood and Marginal Likelihood Estimation of Semiparametric Generalized Linear Models. *J R Stat Soc Series B Stat Methodol*, 73, 3–36.

Zizka, A., Silvestro, D., Andermann, T., Azevedo, J., Duarte Ritter, C., Edler, D., *et al.* (2019). CoordinateCleaner: Standardized cleaning of occurrence records from biological collection databases. *Methods Ecol Evol*, 10, 744–751.

**Marjakangas, Dalsgaard & Ordonez**

**Supplementary material of two appendices to paper “Fundamental interaction niches: towards a functional understanding of ecological networks’ resilience”**

**Appendix S2. Additional results for the case study.**

**Table S1.** The relative influence of each (trait) variable in the gbm model.

| **Trait** | **Relative influence** |
| --- | --- |
| Plant family | 19.75 |
| Corolla length | 11.01 |
| Nectar concentration | 10.32 |
| Maximum height | 6.82 |
| Beak length | 6.31 |
| Mean seed mass | 6.20 |
| Handwing index | 5.42 |
| Beak curvature | 5.39 |
| Tail length | 5.29 |
| Body mass | 4.36 |
| Tarsus length | 4.31 |
| Maximum seed length | 4.00 |
| Hummingbird clade | 3.86 |
| Beak depth | 3.07 |
| Floral color | 2.33 |
| Primary habitat association | 1.58 |

**Table S2.** Generalized additive model summaries for modeling plant rewiring potential as a function of plant species richness and the interaction of grid cell centroid latitude and grid cell centroid longitude. Deviance explained = 82.1%.

|  | **Estimate** | **SE** | **t** | **P-value** |
| --- | --- | --- | --- | --- |
| **Intercept** | 0.901 | 0.001 | 952.700 | <0.001 |
|  | **Expressive degrees of freedom** | **Reference degrees of freedom** | **F** | **P-value** |
| **s(Plant species richness)** | 8.883 | 8.995 | 47.590 | <0.001 |
| **s(Centroid longitude, Centroid latitude)** | 28.994 | 29.00 | 1428.130 | <0.001 |

**Table S3.** Generalized additive model summaries for modeling humming rewiring potential as a function of plant species richness and the interaction of grid cell centroid latitude and grid cell centroid longitude. Deviance explained = 89.9%.

|  | **Estimate** | **SE** | **t** | **P-value** |
| --- | --- | --- | --- | --- |
| **Intercept** | 0.698 | 0.001 | 680.000 | <0.001 |
|  | **Expressive degrees of freedom** | **Reference degrees of freedom** | **F** | **P-value** |
| **s(Hummingbird species richness)** | 8.948 | 8.999 | 681.600 | <0.001 |
| **s(Centroid longitude, Centroid latitude)** | 28.812 | 28.998 | 535.700 | <0.001 |


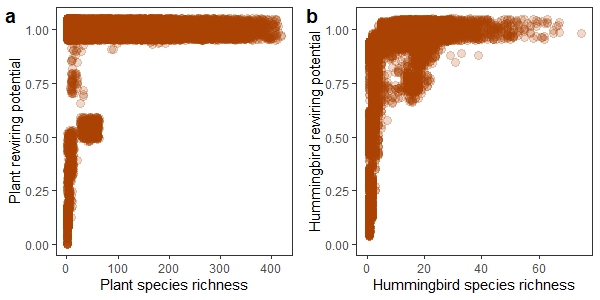


**Figure S1.** Relationship between plant (panel a) and hummingbird (panel b) rewiring potential and their species richness in 0.5x0.5 degree grid cells in the Americas. The plots are based on raw data and data points have been slightly jittered for illustrative purposes.


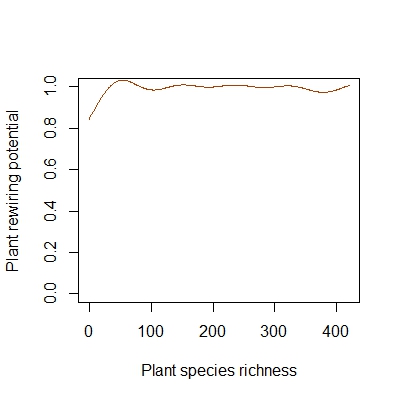

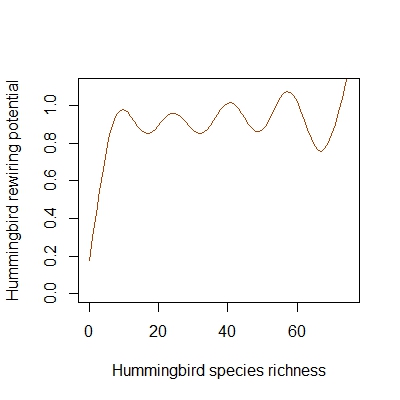


**Figure S2.** Relationship between plant (left panel) and hummingbird (right panel) rewiring potential and their species richness in 0.5x0.5 degree grid cells in the Americas. The plots are based on generalized additive model fit (Tables S2-3).
